# Maintaining Warp Speed: Policy Requirements for a Just-in-Time, Capability-Based, Scalable Medical Countermeasure Research and Development Enterprise

**DOI:** 10.1089/hs.2022.0134

**Published:** 2023-07-18

**Authors:** Colin N. O'Leary, Julia Barnes-Weise, Kendall Hoyt, Margaret Bourdeaux

**Affiliations:** Colin N. O'Leary is a PhD Candidate, Program in Virology, Harvard Medical School, Boston, MA.; Julia Barnes-Weise, JD, CLP, is Executive Director, Global Health Innovation Alliance Accelerator, Durham, NC.; Kendall Hoyt, PhD, is an Assistant Professor, Department of Medicine, Geisel School of Medicine at Dartmouth, and Faculty Director, Pandemic Security Project, Dickey Center for International Understanding, Dartmouth College; both in Hanover, NH.; Margaret Bourdeaux, MD, MPH, is Research Director, Program in Global Public Policy, Department of Global Health and Social Medicine, Harvard Medical School, Boston, MA, and Faculty Fellow, Harvard Kennedy School Belfer Center for Science and International Affairs, Cambridge, MA.

**Keywords:** Medical countermeasures, PHEMCE, Operation Warp Speed, Pandemic preparedness, Biodefense R&D, BioShield

The rise in the incidence of emerging, reemerging, and deliberate disease outbreaks over the past 20 years highlights the threat posed by global catastrophic biological risks (GCBRs).^1^ Since the inception of Project BioShield, the US government has attempted to coordinate the research and development (R&D) of medical countermeasures (MCMs) against a variety of biological, chemical, radiological, and nuclear threats. These efforts led to the development of the MCM enterprise, incorporating numerous agencies, nonprofit and academic groups, and industry partners.

Despite substantial federal investment in MCM development, efforts to speed MCM development against GCBRs have not escaped the cycle of panic and neglect that defines pandemic preparedness and response efforts. Prior to the COVID-19 pandemic, the majority of MCMs against biological threats that achieved licensure after receiving support from the US Biomedical Advanced Research and Development Authority (BARDA) focused on known threats with a recent history of health emergencies: influenza, Ebola virus, and Zika virus.^2^ These investments highlight the reactive nature of BARDA's approach. Even when countermeasures against these pathogens were developed, they were not necessarily paired with production at scale nor developed in time to influence the epidemic trajectory.^3^

The COVID-19 pandemic fundamentally changed this landscape, with the federal government launching Operation Warp Speed (OWS), a centrally led effort to rapidly develop MCMs—particularly vaccines—to confront an urgent and ongoing threat. Vitally, OWS paired the development of these vaccines with simultaneous manufacturing scale-up, enabling vaccines to be widely available shortly after receiving emergency use authorization.^4^ Although this was done at significant cost and at risk, it demonstrated the feasibility of an MCM development and production strategy against a previously unknown threat in the heat of a crisis.

In the summer of 2020, we launched the Task Force on Medical Countermeasures: Partnering for the Public Good to identify shortcomings in the existing US approach to the rapid research, development, and procurement of MCMs; capture the lessons from OWS; and propose a new approach to MCM R&D that would overcome the limitations of the panic and neglect approach to investment. The Task Force was composed of 16 members from a diverse array of backgrounds, including academia, biotechnology, pharmaceutical, public-sector agencies, philanthropy, and advocacy organizations that promote equitable global access to medications. The Task Force convened 4 times, and each Task Force member was interviewed individually over the ensuing 12 months, producing a final report in the fall of 2021. Consultation with Task Force members has continued as we have engaged policymakers to discuss evolving MCM R&D legislation. Below, we summarize the deliberations of the Task Force, including how it characterized the conventional US approach to MCM R&D, the lessons learned from OWS, and the Task Force's proposed framework for a US MCM R&D capability that can rise to confront future pandemic challenges.

## The Problem of the “Conductorless Orchestra”

The federal MCM program is overseen by the Assistant Secretary for Preparedness and Response (ASPR) within the US Department of Health and Human Services (HHS). ASPR chairs the Public Health Emergency Medical Countermeasures Enterprise (PHEMCE), which provides strategic guidance and coordination across agencies involved in developing and stockpiling MCMs within the Strategic National Stockpile. These MCMs are then available to be used “just in case” of an emergency involving a specific pathogen.

Despite ASPR existing to coordinate the MCM enterprise, there is evidence of a “conductorless orchestra” within the PHEMCE, in which individual agencies often performed excellent work but failed to coordinate with each other. For example, both the National Institute of Allergy and Infectious Diseases (NIAID) and BARDA engaged Moderna regarding different projects. NIAID worked with Moderna to demonstrate the versatility of their mRNA vaccine platform, and BARDA supported Moderna's development of a specific Zika virus vaccine.^5,6^ Despite both seeking to leverage mRNA vaccine technology, there is little evidence of coordinated engagement with Moderna by these agencies to advance mRNA vaccine technology.

Further evidence of failures to coordinate within the PHEMCE can be drawn from the federal response to mpox. Despite investment in the smallpox vaccine developed by Bavarian Nordic and purchase of doses for the Strategic National Stockpile, there were delays in making stockpiled doses available. ASPR rapidly worked to negotiate contracts to “fill and finish” the vaccine for use, which could have been negotiated in advance.^7^ Likewise, the procurement of vaccine doses depended on a facility that had not been inspected by the US Food and Drug Administration, another PHEMCE partner agency, meaning that doses from that facility could not be used in the United States until late July, despite being a critical component of the US response.^8^

The lack of a clear conductor for PHEMCE made it difficult to coordinate the rapid development of MCMs in real time during the COVID-19 pandemic. While assessments report that in the years before the COVID-19 pandemic, “PHEMCE developed valuable and deliberative processes,” the response to the pandemic required timely decisionmaking.^9^ A former ASPR criticized the PHEMCE as “not [reflecting] the urgency needed” and claimed that “PHEMCE proceedings created security vulnerabilities.”^10^ Considering these challenges, OWS, not PHEMCE, became the primary federal vehicle for the development of MCMs against COVID-19.

## The Lessons of Operation Warp Speed

OWS was designed to integrate the value chain for MCM development, bringing vaccine candidates from R&D to deployment in under a year. As previously noted, OWS used a nontraditional development strategy in which large-scale manufacturing was initiated in parallel to preclinical development and clinical trials.^4^ This approach demonstrated that a platform technology, such as mRNA vaccines, could be used to rapidly develop an MCM against a previously unknown threat.

OWS benefited from a centralized organizational structure in which there were leaders identified for managing the development of specific products within the OWS portfolio.^11^ These leaders were empowered to make decisions to move forward through the various stages of vaccine development, including rapidly releasing the necessary funding to achieve movement. Our discussions, which were facilitated by the Task Force on MCMs and the Massachusetts Coalition for Pathogen Readiness, revealed that industry leaders and vaccine developers found the centralized leadership structure of OWS preferable to the fragmented PHEMCE structure.

Despite the successes of OWS, we identified 3 pitfalls: the initiative was time limited, reactive, and COVID-19 specific. Following the development of the initial vaccines against COVID-19, OWS transformed its mission from the R&D of COVID-19 vaccines to procurement and distribution. As a result, it failed to account for future needs in the ongoing effort to contain COVID-19.

The urgent assembly of OWS leveraged several billions of dollars in advance purchase agreements (APAs). At the time, these were contracts that could be rapidly negotiated to provide financing for vaccine development. However, there remain numerous issues with APAs negotiated as part of OWS—particularly as it relates to expanding access to MCMs or facilitating substantial increases in production. Importantly, the negotiation of APAs that lacked terms governing vaccine development substantially reduced federal control over the future development of the vaccine, including future boosters, by failing to contract across the full MCM lifecycle.^12^

The failure to mount a serious federal effort to develop and approve the next generation of COVID-19 vaccines represents the weaknesses of a time-limited and reactive approach to MCM development against a pandemic threat. Although BARDA's Beyond the Needle initiative to develop alternative vaccine approaches is regularly highlighted (including efforts to generate an mRNA-based intranasal influenza vaccine), no intranasal COVID-19 vaccines are highlighted in BARDA's investment portfolio, despite their documented potential.^13,14^ If OWS R&D activities had been neither time limited nor solely reactive, accelerated development of second-generation COVID-19 vaccines could have been a critical component of BARDA's vaccine portfolio. Instead, despite calls for an OWS equivalent for intranasal COVID-19 vaccines, the development of next-generation vaccines progressed more slowly, and they may not be available for production at scale or distribution for a long time, if at all.^15^

The American Pandemic Preparedness Plan proposes a robust research agenda for “transforming our medical defenses.”^16^ Likewise, the Biden administration made securing funding for the development MCMs a critical component of its budget request to Congress.^17^ This strategy is centrally coordinated through a steering committee housed within the Office of Science and Technology Policy alongside a directorate within the National Security Council staff. However, recent progress reports on the American Pandemic Preparedness Plan highlight the disparate and disjointed nature of investments, focusing on individual agencies bringing specific products forward, not building cohesive capabilities.^18^ Although strategies have been launched by various agencies, including the *BARDA Strategic Plan* and the *NIAID Pandemic Preparedness Plan*, they seldom refer to each other.^19,20^

Noticeably absent from recent announcements is an effort to build a lead entity that can leverage the work of the plethora of federal initiatives to facilitate the rapid development of MCMs against emerging, reemerging, and deliberately emerging threats and their production at scale. While changes at HHS, such as the creation of the Administration of Strategic Preparedness and Response, hint that this type of leadership structure for MCM R&D may be emerging, the scope of its mandate and authorities remains unclear.^21^ Likewise, efforts to reform the PHEMCE are ongoing and subject to several reviews.^9^ It remains to be seen what this group will look like in the future.

## Reimagining Strategy for the Medical Countermeasures Enterprise

The Task Force identified several key priorities for delivering MCMs with speed and at scale.^22^ Such an effort would involve the identification and maintenance of a portfolio of MCM capabilities, the alignment of public-private partnerships to support capability development, and the use of contracts with terms designed to facilitate MCM development both during and in the absence of a public health emergency. Importantly, these functions would be centralized under the leadership of a single official with the authority and capital available to rapidly make decisions and coordinate with global partners.

The biotechnology revolution enables a shift from a threats-driven MCM development approach to one focused on pathogen-agnostic or broad-spectrum capability development. An example of this capability is mRNA vaccine platforms, which can be redesigned to account for new viral variants or new threats—although they may not be universally protective. Given the diverse range of infectious disease threats, it is more feasible to develop MCM capabilities that can be broadly applied, such as new platforms, versus predefined individual projects against the near limitless number of pathogen threats.

The diversity of pathogen threats highlights the need for a diverse portfolio of MCM capabilities. Certain vaccine and therapeutic capabilities may prove more effective for certain pathogens than others. For example, intranasal vaccine capabilities may be more efficacious against respiratory pathogens than traditional intramuscular administration. Conversely, intramuscular administration may be more suitable for vaccines and therapeutics targeting bloodborne pathogens, such as Ebola virus. At the intersection of this diverse range of pathogens and a diverse portfolio of capabilities is a potentially robust research effort. The development of MCM capabilities must be paired with basic and translational scientific research to identify MCM targets, correlates of protection, and animal models for preclinical MCM validation.

Building successful public-private partnerships is critical to the development of MCMs, which feature unique market structures and costs relative to other medical products. Biomedical innovation and product development is capital intensive, and there is a requirement for sufficient return on investment to justify the opportunity cost of MCM development over that of other medical products. To that end, federal investment must be designed to add value synergistically to industry partners, using tailored incentives to facilitate ongoing participation.

Underlying strong public-private partnerships are flexible contracts governing the conduct of the partners before, during, and after a public health emergency. This is especially important considering the use of rapidly negotiated APAs and their impact on global vaccine equity, as well as development of variant-specific and next-generation vaccines. Negotiating the provisions of these contracts in advance with the flexibility to govern the entirety of the MCM development value chain would increase public-sector leverage while reducing private-sector costs and risks.

Finally, centralized leadership that incorporates integrated research practices across the value chain was previously a feature of MCM development. During World War II, vaccine R&D programs featured single program officers who managed the full lifecycle of development for a particular vaccine.^23^ OWS, as noted, used a similar model, with officials assigned to individual vaccine candidates and managing their progression through development, regulatory smoothing (the process of different government regulatory agencies reaching common standards for product approval), and the release of funding to support development and manufacturing.^11^ This centralized leadership would provide a single point of contact for industry partners, as well as a single point of contact for global partners engaged in MCM development.

## Facilitating the Next Generation of Medical Countermeasure Capabilities

Although the proposed investment in pandemic preparedness and reducing the threat of GCBRs by the Biden administration is welcome, the MCM enterprise has an opportunity to fundamentally realign its research, development, manufacturing, and procurement strategy to better prepare the world to respond to GCBRs. In a world of diverse threats, the predominantly product-based focus targeting disparate disease indications employed by the PHEMCE must be reevaluated. Although developing MCM products against known threats is important, embracing a capability-based approach will facilitate the development of an MCM enterprise prepared to respond to diverse GCBRs, particularly viral threats.

Several benchmarking proposals are discussed below that can be brought forward to implement the Task Force's recommendations on delivering MCMs “just in time” to respond to an emerging pathogen threat. This is an alternative to the traditional “just-in-case” approach taken by the PHEMCE, wherein specific MCMs against known pathogens are produced and stockpiled.

### A Strategic Medical Countermeasure Portfolio

#### Develop a “Just-in-Time” MCM Portfolio

Drawing from the success of OWS, the just-in-time MCM capability portfolio would include key early-stage research, capabilities, and technologies that fit key criteria necessary for a just-in-time MCM. Maintaining a portfolio of capabilities, even redundant ones (ie, multiple vaccine delivery platforms or multiple at-home diagnostic modalities), is critical for reducing the risk of failure. Capabilities and technologies selected for inclusion should fit a series of key criteria:

- *Programmability*: While not truly “pathogen agnostic,” these capabilities should be applicable to multiple pathogens and, if necessary, rapidly reprogrammed to address emerging pathogen variants. Alternatively, these capabilities should be broad in their applicability to pathogens (ie, pan-respiratory viruses).
- *Scalability*: The capabilities should demonstrate an ability to be scaled rapidly for production, consistent with a baseline manufacturing capability equivalent to HHS Technology Readiness Level 8, which would indicate production capability on a scale sufficient for Phase 3 clinical trials.
- *Flexibility*: To sustain a manufacturing base in the absence of a public health emergency, the capabilities should have multiple uses (ie, vaccine capability production facilities should be able to produce vaccines for endemic pathogens or other products; diagnostic platforms should be reconfigured for use to diagnose nonpandemic pathogens).

#### Facilitate Long-Term Planning and Accountability

Of particular importance in developing this capability is the need to address concerns regarding priority setting with PHEMCE and the lack of accountability within ASPR.^9^ Developing MCM capabilities requires a shift in priority setting from the threat-based approach currently used by PHEMCE to a capability-based approach. Likewise, documented instances in which the ASPR was allowed to pursue priorities with limited oversight highlight a need for an independent evidence-based advisory body that would inform portfolio priorities and hold leaders to account. Drawing upon models of outside peer review, such as the Decadal Survey process that advises the National Aeronautics and Space Administration's research portfolio, would establish an independent source of strategic planning and create a high-level advisory body to which leaders investing in the MCM R&D portfolio would be accountable. Partnerships between a just-in-time MCM authority and the National Academies of Sciences, Engineering, and Medicine represent a route to creating such a high-level advisory body. Such a panel can be aligned with the proposed PHEMCE advisory committee proposed by the National Academies of Sciences working group, but with an independent charter and purpose in addition to reviewing the traditional MCM development pipeline.^9^

#### Scout and Source Novel Technologies

Other successful enterprises that support the deployment of evolving advanced technologies, either for strategic or corporate needs, such as In-Q-Tel, constantly survey the R&D ecosystem to identify promising technologies and capabilities for inclusion in their investment portfolios. To ensure that the technologies and research needed for rapid development are available to the authority responsible for building a just-in-time capability, that authority should have the ability to identify, source, and negotiate terms of acquisition from providers of research, development, and manufacturing assets deemed necessary to the capability.

Currently, scouting efforts are conducted under the auspices of BARDA's Division of Research, Innovation and Ventures. A just-in-time MCM development authority could leverage this network and other existing networks of entrepreneurs in scouting and sourcing capabilities. However, unlike BARDA's advisory network, the purpose of this function would be to actively identify candidates for portfolio inclusion and investment.

### Centralized Capability Leadership

#### Create a Single Coordinating Office

A critical lesson learned from OWS is the importance of a centrally organized structure that is empowered to rapidly make decisions. This is vital for portfolio management in an emergency: the ability to make rapid decisions on the structure of the portfolio and shift resources accordingly is key to ensuring the products with the highest likelihood of success moving forward. This became apparent with OWS, in which less promising vaccine candidates were removed from the portfolio as other candidates accelerated through the development pipeline.

Additionally, central coordination is vital given the diverse number of government agencies developing MCMs. A key component of OWS was the integration of the US Department of Defense and its research programs into the development of COVID-19 MCMs alongside those housed within HHS. As an example of the importance of integration, the Joint Science and Technology Office for Chemical and Biological Defense (within the US Department of Defense) is developing predictive capabilities for vaccine-platform technologies, which would be of significant import to a just-in-time capability and should be developed and utilized via a central coordination mechanism.^18^

#### Embrace Organizational Flexibility

The Defense Advanced Research Products Agency model of R&D is characterized by substantial organizational flexibility, which is a key component in maintaining a just-in-time MCM R&D capability. This flexibility “[allows] agencies to respond more quickly to changing technological conditions” via the flat organizational structures that facilitate direct reporting to leaders and hiring outside traditional government streams.^24^ These arrangements bring in new ideas and allow fresh eyes to evaluate priorities and approaches. As previously noted, dynamic portfolio management demands both the ability to scout and source novel capabilities and the ability to remove underperforming and outdated capabilities from the portfolio.

#### Facilitate Global Partnerships

MCMs are most effective when they are deployed where outbreaks first occur, preventing them from spreading and becoming pandemics. This means MCM manufacturing and deployment is a global endeavor, as decisions about how to scale production and where to deploy MCMs first need to be made irrespective of borders. Although the global governance architecture necessary to manage the complex undertaking of pandemic preparedness is being negotiated across many stakeholders, directors of MCM R&D innovation pipelines and portfolios are an important group that should contribute to these negotiations. While specific MCM R&D portfolios do not need to be tightly coordinated—variations in focus and some redundancies are desirable—directors of MCM R&D innovation portfolios need mechanisms through which they can come together to decide which populations should be prioritized in terms of access to MCMs and under which conditions. The COVID-19 Vaccines Global Access (COVAX) facility, a global collaboration structured to invest in MCMs and distribute them globally, was an important, if under-supported and under-resourced, step in this direction. An analogy for this type of negotiated process can be found in the security sector, where multilateral security organizations ensure their members have the necessary means and production tools to produce defenses if they are attacked.

### Realigned Public–Private Partnerships

#### Use “Pivot-and-Scale” Contracting

Central to building just-in-time MCM capability and addressing the failures of OWS is ensuring that investments made before a pandemic can be leveraged during a pandemic by pivoting and scaling production. This involves using flexible contracting approaches that allow for pivoting of programmable MCM capabilities between pathogen threats and directing the scale at which it must be produced. Such flexible contracts can be developed using an “other transaction authority,” or OTA, which is a legal procurement instrument that is not required to comply with the rules outlined in the Federal Acquisition Regulation. Use of such an instrument must be paired with best practice inclusion of rights to share resulting products with other countries that would enable more leverage globally.

Such terms would leverage early-stage government investment for late-stage benefit. Early-stage investments would be used for a variety of purposes that increase value across an industry partner's portfolio (ie, federal investment in improving mRNA delivery would benefit all mRNA products). Contracts for these early-stage investments would contain clauses obligating the portfolio partner to pivot their production to a candidate MCM and scale its manufacturing by diverting manufacturing from other products during a public health emergency. The terms of these “pivot-and-scale” clauses could be tailored to the type and level of risk of the specific class of pathogen and modified as our understanding of pathogen risk changes over time. These contracts would contain a predetermined formula for agreement on the prices charged for MCMs produced via this mechanism and terms addressing the possibility of further investment in manufacturing.

#### Align Incentive Structures

As discussed, early-stage investments in the R&D of platform technologies create value across the entire portfolio of an industry partner using that platform. Enhancing portfolio value is critical for an industry partner, which relies on private capital and sustained private investment. However, it also creates broader value for governments, which have a fundamental interest in developing advanced technologies. This is particularly true in the biotechnology space, which has been noted as having substantial economic potential.

To this end, the leadership of a just-in-time capability authority should work with other agencies managing the National Biotechnology and Biomanufacturing Initiative to align incentive structures for participating in federally funded programs. Linking federal funding for R&D capital investment in manufacturing and other areas in this space to participation in MCM development programs creates incentives that benefit public and private partners in confronting key challenges.

### Sustained and Constant Operations

#### Enable Sustained Manufacturing and Regular Testing of Capabilities

Despite a longstanding partnership between NIAID and Moderna to develop vaccine candidates, COVID-19 represented the first test of that partnership.^25^ While the partnership was fortunately successful, this was in no way guaranteed.^26^ Moving forward, capabilities included in the portfolio should be regularly tested and manufacturing processes continuously improved by facilitating manufacturing challenges and continuous small-batch manufacturing of MCM candidates. This would allow the capabilities of industry partners to be regularly tested and their ability to pivot manufacturing, produce novel MCM candidates, and, where possible, generate preclinical and clinical data assessed for both their own and the government's benefits.

Importantly, this assessment would also allow for continuous review of the portfolio's performance and that of the individual capabilities contained within it. With regular challenges, modeled on the Coalition for Epidemic Preparedness Innovations' 100-day challenge, the leadership of a just-in-time MCM capability could regularly review and resolve successes, failures, and unexpected issues.^27^ Given the uncertainties involved in pathogen behavior and the difficulties in predicting which pathogens will flame out and which truly have pandemic potential, it is critical to exercise, assess, and revise pivot-and-scale triggers and capabilities. Moreover, 100-day challenges, wherein a new MCM is successfully developed, may lead to new therapies that treat common infectious pathogens, incentivizing investment in MCM R&D even in the absence of a health emergency.

#### Provide Long-Term Financing

Vital to testing capabilities is the ability to finance tests. Before the COVID-19 pandemic, Moderna planned to conduct a “stopwatch” drill in collaboration with NIAID to test its capability to rapidly produce a clinical-grade vaccine product. However, the funding for this was not readily available and was subject to the approval of Moderna's board of directors.^25^ Likewise, even as COVID-19 emerged, early funding for the initial development of a candidate vaccine had to be raised rapidly from several sources. This is not a sustainable model for MCM innovation or development.

Project BioShield made funding available for biodefense purposes and MCM development over an initial 5-year period. Recent proposals put forward by the Biden administration build on this model by including appropriations over 5 years.^17^ To ensure that funding is available for fulfilling long-term contracts and testing capabilities, financing over a 10-year horizon is necessary. Long-term financing might be achieved by attaching this funding to a legislative vehicle that enjoys wide bipartisan support, such as US Department of Defense appropriations, or using mandatory spending (ie, Medicare, Social Security). Other models, like creating a semi-independent MCM authority with its own revenue streams in addition to public-sector financing, should be considered. For example, an MCM R&D authority could charge a small percentage on the commercial benefits of its investments.

Although additional funding for the MCM enterprise would be preferable, failure in recent months to pass supplemental appropriations to continue MCM development against COVID-19 highlights potential political constraints on increasing funding. Alternatively, opportunities to spur collaboration between federal agencies and develop joint capabilities that leverage spending across departments should be assessed. Additionally, modifying appropriations structures to allow greater flexibility in spending (versus requiring spending for specific disease categories) would enable the MCM enterprise to embrace pathogen-agnostic MCM capabilities.

## Conclusion

Building on the successes and learnings from the COVID-19 pandemic, now is an important time to refocus US MCM R&D strategies. The MCM investment strategies used by public health agencies can be transformed to build a just-in-time MCM capability for future threats. It is critical to advance not only the R&D necessary to mitigate the threat of GCBRs but also the governance mechanisms needed to ensure that those advances can become MCMs that can alter the course of the next epidemic.
